# Effect of Different Influent Conditions on Biomass Production and Nutrient Removal by Aeration Microalgae Membrane Bioreactor (ICFB-MMBR) System for Mariculture Wastewater Treatment

**DOI:** 10.3390/membranes11110874

**Published:** 2021-11-14

**Authors:** Yi Ding, Shiyuan Wang, Hang Ma, Binyu Ma, Zhansheng Guo, Hong You, Junxue Mei, Xuguang Hou, Zhenlin Liang, Zhipeng Li

**Affiliations:** 1 Marine College, Shandong University, Weihai 264209, China; dingyits@126.com (Y.D.); guozhansheng@sdu.edu.cn (Z.G.); meijunxue@sdu.edu.cn (J.M.); richardhoukk@163.com (X.H.); liangzhenlin@sdu.edu.cn (Z.L.); 2 State Key Laboratory of Urban Water Resources and Water Environment, School of Marine Science and Technology, Harbin Institute of Technology, Weihai 264200, China; Wang13792541713@163.com (S.W.); 19b954023@stu.hit.edu.cn (H.M.); a958444903@foxmail.com (B.M.); youhong@hit.edu.cn (H.Y.)

**Keywords:** microalgae membrane bioreactor, mariculture wastewater, nutrient removal, biomass production, different influent conditions

## Abstract

The nutrient removal and biomass production of the internal circulating fluidized bed microalgae membrane bioreactor (ICFB-MMBR) was studied under different cultivation modes, influent TOC, influent pH, and influent N/P. *Platymonas helgolandica tsingtaoensis* was used as the biological source. The growth of *P. helgolandica tsingtaoensis* and the removal efficiency of pollutants in the mixotrophy culture mode were improved compared with other culture modes. With the increased influent TOC, the average growth rate of *P. helgolandica tsingtaoensis* increased, and ammonia nitrogen and total phosphorus removal rate were improved. The *P. helgolandica tsingtaoensis* growth rate and nutrient removal efficiencies at the influent pH of 8 were the best among the different influent pH values. As the influent N/P ratio increased from 5 to 20, the *P. helgolandica tsingtaoensis* growth rate and pollutant removal rate increased gradually. When the influent N/P ratio was higher than 20, the *P. helgolandica tsingtaoensis* growth rate and pollutant removal rate tended to be stable and did not significantly change with the increase of influent N/P ratio. At the proper influent conditions, the high *P. helgolandica tsingtaoensis* biomass and nutrient removal efficiency could be obtained in the microalgae membrane bioreactor, which could provide a theoretical basis for the application of the system for wastewater treatment.

## 1. Introduction

In recent years, the mariculture industry has tended towards an intensive, high-density, and high-yield aquaculture model, which has led to the increasingly prominent problem of marine aquaculture environmental pollution [1,2,3]. Mariculture wastewater usually contains a large amount of pollutants from animal excrement and feed residues, which are very harmful to the environment and aquatic organisms [4], and the salinity for mariculture wastewater is similar with that of seawater. How to design and optimize the process with good treatment efficiency has been a research hotspot in mariculture wastewater treatment [5,6,7].

Microalgae exhibited great versatility as energy sources, and many studies have suggested the combination of algal biomass production with wastewater treatment [8]. The treatment of mariculture wastewater by microalgae is an emerging and sustainable technology [9,10,11]. It could effectively accumulate microalgae biomass with the pollutants’ removal in wastewater [12]. The harvested microalgae could be used as aquatic animal bait, human health food, bioenergy raw materials, skin care raw materials, and so on, and many studies have shown that many microalgae have a certain salt tolerance [13,14]. In the past, researchers have made great efforts to develop efficient and economical microalgae technology for the removal of nitrogen and phosphorus in wastewater [15,16].

*Platymonas helgolandica tsingtaoensis,* found widely in saline/brackish waters in the world, are mainly used as a food source in marine aquaculture [17]. It has been reported that some microalgae were supplementary sources of nourishment for aquatic animals, for example, essential amino acids, vitamins, and some essential fatty acids [18,19], and this nourishment could enhance immune function [20]. Cultivation of *Platymonas helgolandica* in the rearing water of white shrimp conferred positive effects for shrimp aquaculture, considering water quality and growth performance [21]. The present study demonstrated that the marine green alga *P. helgolandica var. tsingtaoensis* was capable of H_2_ photoproduction when treated by the protonophore uncoupler [22]. Utilizing marine microalgae for the biological production of H_2_ gas was a promising approach for meeting future energy needs. Therefore, *Platymonas helgolandica tsingtaoensis* was used as the biological source in this study.

As a kind of biological treatment method, membrane bioreactor (MBR) has the dual characteristics of biological treatment and membrane separation [23,24]. Compared with the traditional activated sludge method, MBR has the advantages of high effluent quality, a high degree of automation, and a small floor area. However, membrane fouling is still the main obstacle restricting the wide application of membrane technology [25,26,27]. When the MBR was used in saline wastewater treatment, the salinity had an effect on the microbial activity, and then decreased the removal efficiency of ammonia nitrogen [28,29,30]. Combined with microalgae, the MBR could completely intercept microalgae cells to ensure biological activity, and independently control solid retention time (SRT) and hydraulic retention time (HRT) [31], which made the microalgae membrane bioreactor have a higher biomass accumulation efficiency. The microalgae membrane bioreactor could absorb nitrogen and phosphorus in wastewater through microalgae growth and metabolism [32,33].

In a previous study, a novel microalgae membrane bioreactor with an internal circulating fluidized bed was constructed to efficiently degrade pollutants and enrich microalgae in order to realize the reuse of marine aquaculture wastewater, and slight and slow membrane fouling was observed [34]. This process of microalgae membrane bioreactor with internal circulating fluidized bed was affected by many factors, including wastewater characteristics, operating factors, and environmental factors [11,35].

According to different culture conditions and wastewater components, microalgae could be cultured under photoautotrophic or heterotrophic conditions, and some species could be cultured under mixotrophy condition. Photoautotrophy microalgae used inorganic carbon to grow and metabolize under light, while heterotrophic microalgae used organic carbon to grow and metabolize in the dark [36,37]. In mixotrophy culture, microalgae could absorb both inorganic and organic carbon for photosynthesis and other metabolic pathways [38]. Most algal species were able to synthesize organic macromolecules via photosynthesis [39], and several studies were concentrated on autotrophic growth in which inorganic carbon was used as the main carbon source. In the process of mariculture, organic matter in mariculture wastewater would inevitably be produced. The introduction of organic matter in wastewater was conducive to the formation of mixotrophy culture mode of microalgae. In view of the large fluctuation of pollutant concentration in mariculture wastewater, the biochemical reaction of microalgae might be different with the different concentration of organic matter.

Because pH played an important role in the proliferation of microorganisms, it affected the microalgae degradation ability for pollutants in wastewater. The high pH has been shown to inhibit photosynthesis [40].For the photosynthesis, pH levels could rise to above 9.0, as CO_3_^2−^ and HCO_3_^2−^ were consumed to produce CO_2_ for cell growth and OH^-^ ions were left in excess [41]. In addition, the pH values could be adjusted to the suitable range, which was verified to benefit the growth of algae [42]. In the actual aquaculture project, the pH in mariculture wastewater was different owing to different aquaculture modes and biological species. The N/P ratio was correlated with the rate of cell proliferation in marine microalgae. The preference for nitrogen and phosphorus varied among marine microalgae [43]. For example, *dinoflagellates* preferred lower N/P ratios, whereas *diatoms* preferred a higher ratio [44]. It had been reported that optimal N/P ratios would vary from 8.2 to 45.0, depending on the ecological conditions [45,46]. Owing to different microalgae species and culture methods, the required N/P ratio was also different.

However, most previous studies have been carried out under suitable or optimal conditions [47,48,49]. This would hinder the scale-up and commercial application of algae cultivation. Thus, how to improve the performance of microalgal cultivation and nutrient removal in wastewater was an urgent issue that needed to be solved. At present, many studies focused on the treatment efficiency of microalgae membrane bioreactor, and few studies have reported on the effect of the operation parameter on the microalgae membrane bioreactor. Therefore, in this study, *Platymonas helgonica tsingtaoensis* was used as an algae species to treat simulated mariculture wastewater by internal circulating fluidized bed microalgae membrane bioreactor. The biomass production and nutrient removal efficiency of microalgae membrane bioreactor were discussed under different culture modes, different influent TOC concentrations, different influent pH values, and different influent N/P ratios. This research could provide a theoretical basis for the application of the microalgae membrane bioreactor process to the treatment of saline wastewater.

## 2. Materials and Methods

### 2.1. Microalgae Membrane Bioreactor System

**Figure 1:**
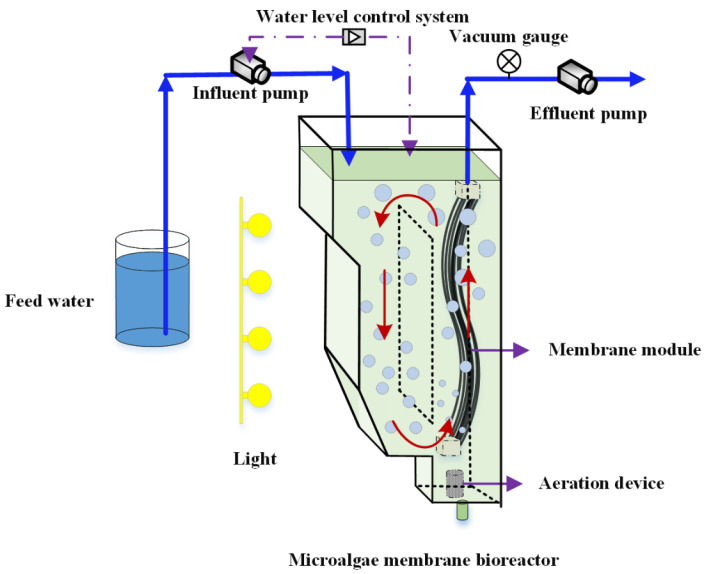
Internal circulating fluidized bed microalgae membrane bioreactor used in the experiment.

The internal circulating fluidized bed microalgae membrane bioreactor was used in this study, as shown in Figure 1. The microalgae membrane bioreactor was made of plexiglass with an effective volume of 3.0 L. The algal mixed liquid in the reactor could realize internal circulation through aeration with the aeration rate of 0.8 L/min at the bottom. The polyvinylidene fluoride (PVDF) hollow-fiber membrane (Motian Co. Ltd., Tianjin, China) was installed in the reactor. The membrane module was placed above the aeration strip which could effectively mitigate the occurrence of membrane fouling by reducing the accumulation of microalgae on the membrane surface with the help of the aeration scouring. The pore diameter of membrane module was 0.03 μm, and the total membrane area was 0.04 m^2^. In the experiment of continuous water inlet, the liquid level in the reactor was controlled by the liquid level controller, the effluent was discharged through the suction of the peristaltic pump, and the time relay was used to control the intermittent effluent (8 min pumping, 2 min stop) to delay membrane fouling. The effluent rate was 3.9 L/m^2^ h, the HRT was 1 d, and the reactor was operated at room temperature. The reactor was operated by removing 0.15 L of the mixed liquor every day to maintain the solid retention time (SRT) of 20 days. The transmembrane pressure (TMP) was recorded by a precision vacuum pressure gauge. Once the TMP reached 30 kPa in the MMBR, the membrane modules were taken out and cleaned. The membrane modules were reloaded into the bioreactors to run after cleaning. During the operation of each stage, the TMP was always below 10 kPa in the MMBR. The slight and slow membrane fouling was not discussed in the manuscript.

### 2.2. Operation of Internal Circulating Fluidized Bed Microalgae Membrane Bioreactor

#### 2.2.1. Source and Culture of Microalgae

The microalgae species used in this study was *Platymonas helgonica tsingtaoensis*, purchased from the Institute of Oceanography, Chinese Academy of Sciences, Qingdao, China. *P. helgonica tsingtaoensis* were inoculated into a conical flask with f/2 culture medium [50]. The culture temperature was about 22 °C, and the culture light intensity was 2000 lx with a culture light and dark ratio of 12 h/12 h. The conical flask was oscillated three times a day to prevent microalgae precipitation.

#### 2.2.2. The Composition of Simulated Mariculture Wastewater

In this study, the internal circulating fluidized bed microalgae membrane bioreactor was used for simulated mariculture wastewater treatment. Organic carbon, inorganic carbon, ammonia nitrogen, and phosphorus sources were provided by glucose, carbon dioxide in air, ammonium chloride, and potassium dihydrogen phosphate, respectively, and were diluted by seawater collected from Huang Hai (Shidao, Weihai, China). The salinity was 3.45%. The main pollutants in wastewater were as follows: TOC 40–120 mg/L, ammonia 5–30 mg/L, and phosphate 3 mg/L. The pH was adjusted and maintained in the set range with 1 mol/L HCl and 1 mol/L NaOH. In addition, a proper amount of trace elements were added: Na_2_EDTA 4.36 mg/L, FeCl_3_·6H_2_O 3.16 mg/L, CuSO_4_·5H_2_O 0.01 mg/L, ZnSO_4_·7H_2_O 0.023 mg/L, CoCl_2_·6H_2_O 0.012 mg/L, MnCl_2_·4H_2_O 0.18 mg/L, Na_2_MoO_4_·2H_2_O 0.07 mg/L, B_1_ 0.1 mg/L, B_12_ 0.0005 mg/L, and Biotin 0.0005 mg/L, among others.

#### 2.2.3. The Experimental Method

This study aimed to explore the treatment efficiency of mariculture wastewater in the microalgae membrane bioreactor under different operating conditions. The experimental arrangement is listed in Table 1. The cultures were bubbled with air to supply CO_2_. The continuous influent mode was adopted to study the effects of different influent TOC concentrations, different influent pHs, and different culture modes on microalgae membrane bioreactor. The batch culture was first used to increase the initial microalgae concentration, and then the membrane filtration was use to study the influence of N/P on the microalgae membrane bioreactor.

Prior to the experiments, the microalgae was cultured for 60 days with the SRT of 20 days. In each reactor, the initial biomass was 0.12 g/L. At present, the reactor was still in operation, and the experiment result for 20 days’ operation was provided. The nutrient removal efficiency and biomass production of microalgae membrane bioreactor under different culture modes (photoautotrophic, heterotrophic, and mixotrophy), different influent TOC concentrations (40 mg/L, 80 mg/L, and 120 mg/L), different influent pH values (6, 7, 8, and 9), and different influent N/P ratios (5, 10, 15, 20, 25, and 30) were discussed.

### 2.3. Water Sample Collection and Determination Method

#### 2.3.1. Collection and Biomass Determination of Microalgae

Owing to the small size of microalgae cells, poor sedimentation performance, and limited density in aqueous solutions, the centrifugal method was used for the collection of microalgae. The microalgae liquid was placed in the centrifuge tube, and the centrifuge speed was set at 5000 rpm with a centrifuge time of 15 min. More microalgae in the algal liquid could be collected, and the biomass of microalgae could be measured by the dry weight method.

#### 2.3.2. Water Quality Analysis Method

In this study, the concentrations of organic matter, total phosphorus, and ammonia nitrogen in the influent and effluent water of the reactor were analyzed. The water sample was taken from influent and effluent water tank, respectively. The water sample was centrifuged at 5000 rpm for 15 min. After centrifugation, the supernatant was filtered by a 0.45 μm membrane, and the filtrate was measured.

The method of spectrophotometer was used to determine the nitrogen and phosphorus concentration of water samples. The total organic carbon was measured by a TOC meter (TOC-5000A, Shimadzu, Kyoto, Japan). All the above analyses were performed in triplicate, and their average values were listed.

## 3. Results

### 3.1. Effect of Different Culture Modes on Microalgae Membrane Bioreactor Perforamence

#### 3.1.1. P. helgolandica tsingtaoensis Growth

**Figure 2:**
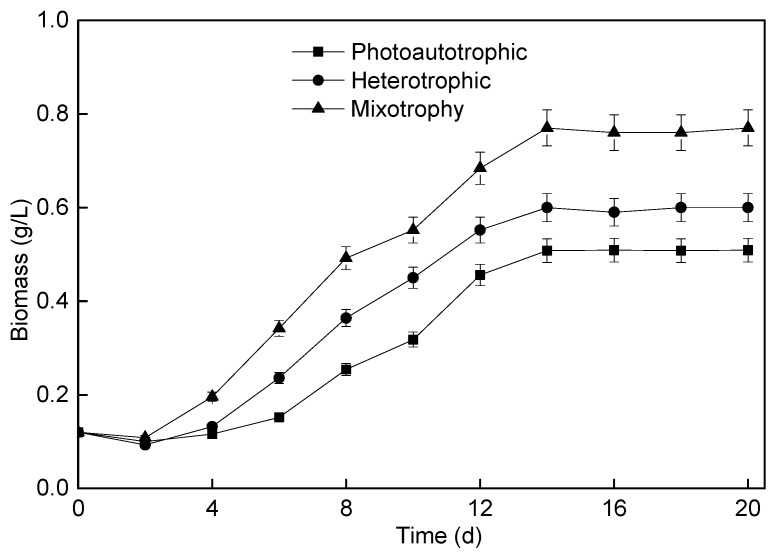
Changes in *P. helgolandica tsingtaoensis* biomass concentration under different culture modes.

During the operation of microalgae membrane bioreactor, the performance of microalgae membrane bioreactor can be better understood through the continuous monitoring of *P. helgolandica tsingtaoensis* biomass concentration. The changes in *P. helgolandica tsingtaoensis* biomass concentration under different culture modes are shown in Figure 2. It could be seen that the change in biomass concentration was closely related to the culture mode.

When the concentration of *P. helgolandica tsingtaoensis* under autotrophic conditions reached 0.46 g/L at day 12, after that, the increased biomass of *P. helgolandica tsingtaoensis* began to slow. This was because the growth rate of *P. helgolandica tsingtaoensis* under autotrophic conditions was limited by light intensity, especially when the concentration of *P. helgolandica tsingtaoensis* was high. The heterotrophic culture *P. helgolandica tsingtaoensis* with ammonia nitrogen as nitrogen source grew faster than the photoautotrophic culture *P. helgolandica tsingtaoensis*. Compared with heterotrophic and photoautotrophic conditions, mixotrophy culture microalgae with ammonia nitrogen as a nitrogen source grew more rapidly. This may be because *P. helgolandica tsingtaoensis* can utilize both organic and inorganic carbon sources under mixotrophy conditions. When ammonia nitrogen was used as a nitrogen source, the *P. helgolandica tsingtaoensis* biomass concentrations of photoautotrophic, heterotrophic, and mixotrophy culture reached 0.51 g/L, 0.60 g/L, and 0.77 g/L, respectively, and the average biological growth rates were 27.71 mg/(L·d), 34.29 mg/(L·d), and 46.43 mg/(L·d), respectively.

When there were both organic and inorganic carbon sources in wastewater, the existence of organic carbon would change the mechanism of microbial photosynthesis and respiration under light. It has been reported that the combination of photoautotrophic and heterotrophic culture can effectively improve the biomass of microalgae [38,51,52]. The mixotrophic growth could be the fastest way to grow algal biomass compared with the autotrophic and heterotrophic growth [53,54]. Therefore, in practical engineering applications, it could be considered to appropriately increase small molecular organics such as glucose to quickly improve the yield of *P. helgolandica tsingtaoensis*.

#### 3.1.2. Nutrient Removal

**Figure 3:**
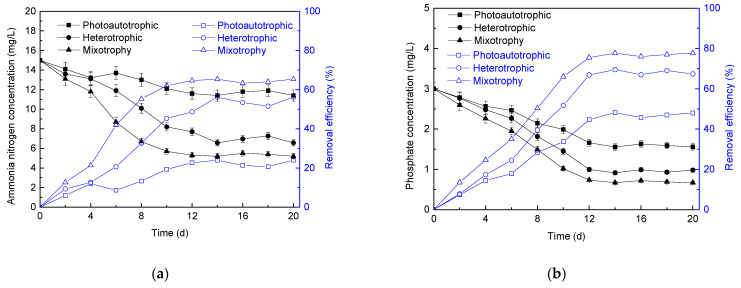
Influence of different culture modes on ammonia nitrogen (**a**) and phosphorus (**b**) removal.

The variation in ammonia nitrogen concentration and removal efficiency in the effluent of microalgae membrane bioreactor with different culture modes is shown in Figure 3a. With the operation of the reactors, the reactors under heterotrophic culture and mixotrophy culture mode showed better effluent quality compared with the photoautotrophic mode, and the ammonia nitrogen concentrations in the effluent were 6.57 mg/L and 5.20 mg/L, respectively.

The variation in phosphate concentration and removal efficiency in the effluent of microalgae membrane bioreactor with different culture modes is shown in Figure 3b. It was observed that the effluent total phosphorus concentrations of the photoautotrophic, heterotrophic, and mixotrophy culture modes were decreased to 1.56 mg/L, 0.92 mg/L, and 0.67 mg/L, respectively. The total phosphorus removal rate could reach 2.33 g/(m^3^·d) under the mixotrophy condition. For the photoautotrophic and heterotrophic culture, the removal rates of total phosphorus were 1.45 g/(m^3^·d) and 2.09 g/(m^3^·d), respectively. Thus, the phosphorus removal rate in mixotrophy culture mode was more than twice that in photoautotrophic culture.

In conclusion, good nitrogen and phosphorus removal efficiency could be obtained by producing high-density mixotrophy microalgae in microalgae membrane bioreactor with ammonia nitrogen as a nitrogen source. The removal efficiencies of ammonia nitrogen and total phosphorus in photoautotrophic microalgae membrane bioreactor were lower than those in heterotrophic and mixotrophy culture membrane reactor. This was because the ATP transfer efficiency of energy absorbed by microalgae under heterotrophic conditions (18%) was higher than that under photoautotrophic conditions (10%) [55]. In photoautotrophic microalgae cells, ATP was produced by mitochondria, and 77% of ATP was used to fix carbon dioxide through the calvin cycle, while the rest was converted to organic compounds [55]. Therefore, the treatment efficiency of photoautotrophic microalgae membrane bioreactor on nitrogen and phosphorus was lower compared with other culture modes.

### 3.2. Effect of Different Influent TOC Concentration on Microalgae Membrane Bioreactor Performance

#### 3.2.1. *P*. *helgolandica tsingtaoensis* Growth

**Figure 4:**
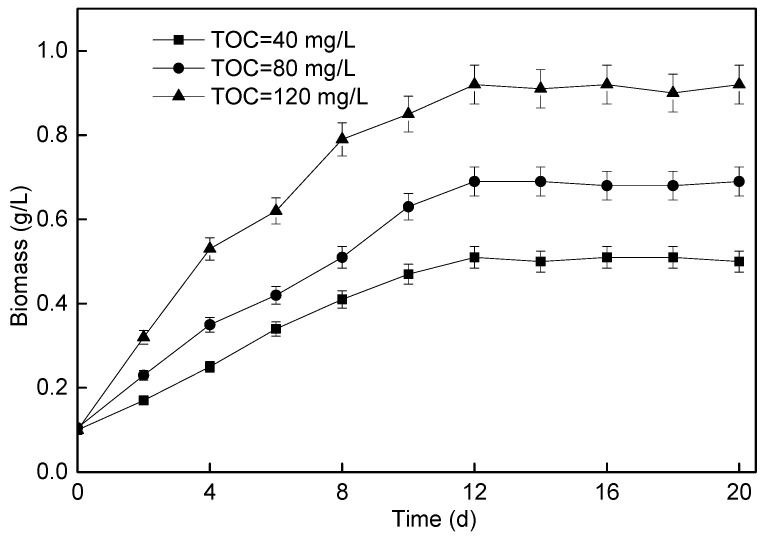
Changes in *P. helgolandica tsingtaoensis* biomass concentration under different influent TOC.

The growth of *P. helgolandica tsingtaoensis* in wastewater with different influent TOC concentrations is shown in Figure 4. It could be seen from the figure that the growth rate of *P. helgolandica tsingtaoensis* in the reactors increased with the increase in influent TOC concentration. When the influent TOC concentration was 40 mg/L, 80 mg/L, and 120 mg/L, respectively, the maximum biomass was 0.51 g/L, 0.69 g/L, and 0.92 g/L, respectively, and the average biomass growth rate was 32.50 mg/(L·d), 47.50 mg/(L·d), and 66.67 mg/(L·d), respectively, which indicated that the concentration of organic matter in wastewater was an important factor affecting the growth of mixotrophy microalgae.

In the growth process of *P. helgolandica tsingtaoensis*, carbon, nitrogen, and phosphorus were mainly needed. When the nitrogen and phosphorus sources in the wastewater were sufficient, the *P. helgolandica tsingtaoensis* biomass normally showed an upward trend with the increase in TOC concentration. Therefore, the supply of organic matter could stimulate the growth of *P. helgolandica tsingtaoensis*. In addition, some researchers also reported that high concentrations of organic matter might also inhibit the growth of microalgae. Liang et al. reported that the optimal glucose concentration for *chlorella* culture was 1% (10 g/L), and inhibition occurred when it exceeded this concentration [56]. Cheirsilp et al. reported that the dry weight of *chlorella* increased when the initial glucose concentration increased from 0 to 10 g/L, but did not further increase when it reached 20 g/L [57]. In this study, when the TOC concentration of wastewater increased from 40 mg/L to 120 mg/L, the growth rate of *P. helgolandica tsingtaoensis* increased, indicating that organic matter could promote the growth of *P. helgolandica tsingtaoensis* in this concentration range.

#### 3.2.2. Nutrient Removal

**Figure 5:**
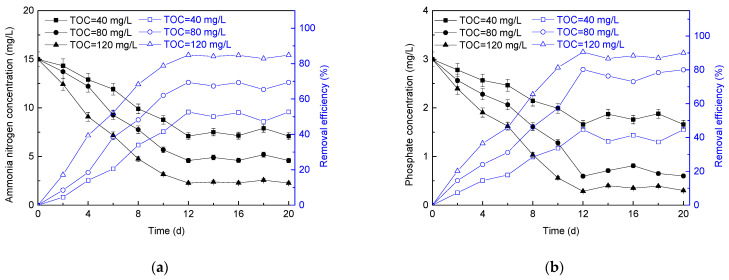
Changes in ammonia nitrogen (**a**) and phosphate (**b**) removal efficiency under different TOC concentrations.

The changes in effluent ammonia nitrogen concentration and ammonia nitrogen removal efficiency under different TOC concentrations during the operation of microalgae membrane bioreactor are shown in Figure 5a. It could be seen that the influent TOC concentration had a significant impact on the removal efficiency of ammonia nitrogen. With the increased in influent TOC concentration, the removal efficiency of ammonia nitrogen in wastewater increased gradually.

When the influent TOC concentration was 40 mg/L, 80 mg/L, and 120 mg/L, respectively, the effluent ammonia nitrogen concentration decreased to 7.10 mg/L, 4.59 mg/L, and 2.27 mg/L, respectively, and the ammonia nitrogen removal efficiencies were 53%, 69%, and 85%, respectively. Compared with the influent TOC concentration of 40 mg/L, the rector with the influent TOC concentration increasing to 120 mg/L can obtain a higher ammonia nitrogen removal efficiency.

The influence of different influent TOC concentrations on the total phosphorus removal efficiency of microalgae membrane bioreactor is shown in Figure 5b. With the continuous operation of the reactors, the microalgae biomass increased to the maximum in the three reactors, and the absorption capacity of pollutants in the wastewater was further enhanced. At this time, the effluent total phosphorus concentration with the influent TOC concentration of 40 mg/L, 80 mg/L, and 120 mg/L decreased to 1.66 mg/L, 0.60 mg/L, and 0.29 mg/L, respectively, and the total phosphorus removal efficiencies were 45%, 80%, and 91%, respectively.

Nitrogen and phosphorus were essential nutrients for the synthesis of some biological substances in algae cells, such as protein, chlorophyll, and nucleic acid. Generally, nutrient uptake by algae cells was considered to be the main way to remove nutrients in microalgae wastewater treatment [58]. It could be seen that, with the increase in TOC concentration, the growth rate of microalgae was accelerated, the absorption capacity of pollutants was enhanced, and the change trend of ammonia nitrogen and total phosphorus in wastewater was similar.

In addition, some chemical processes, such as chemical precipitation and ammonia volatilization, could also remove some nitrogen and phosphorus from wastewater. However, these chemical removal processes were mainly affected by pH changes caused by microalgae growth. It could be seen that the growth of microalgae was the main factor affecting the removal of nutrients in wastewater [59]. Similar to this study, some previous studies also reported that, under the mixotrophy conditions of glucose and other organic substances, owing to the high algae biomass in the mixed nutrition culture, the removal efficiencies of nitrogen and phosphorus were higher than that under the photoautotrophic condition [60].

Gao et al. explored the effects of different TOC/TN on the nutrient removal of *Chlorella vulgaris* under the mixotrophy culture mode, and found that, when TOC/TN increased from 0 to 24, the growth rate of microalgae accelerated and the pollutant removal efficiency increased [13]. It was similar to the experimental phenomenon in this experiment, but the pollutant removal efficiency obtained by Gao et al. was far lower than the pollutant removal level obtained in this experiment. This might be because the continuous culture in this experiment was conducive to maintaining the stable operation of the reactor system, so the development of steady-state microalgae cultivation was more rapid and the removal efficiency of pollutants was strengthened.

#### 3.2.3. TOC Removal

**Figure 6:**
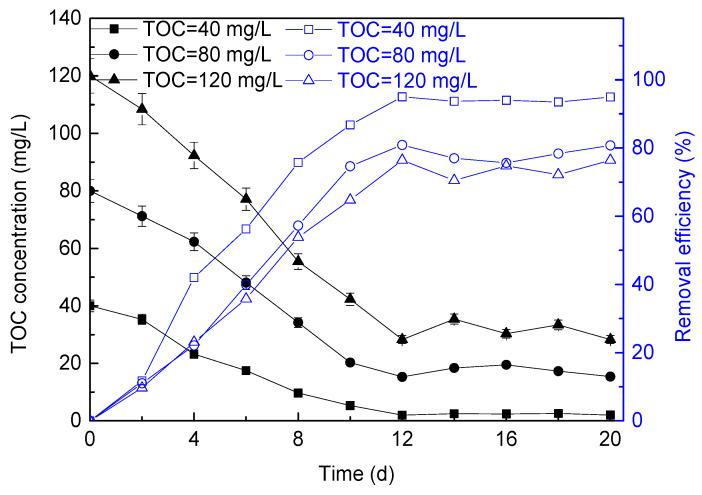
Changes in effluent TOC concentration and TOC removal efficiency under different TOC concentrations.

The influence of different TOC concentrations on TOC removal efficiency of microalgae membrane bioreactor is shown in Figure 6. It could be seen from the figure that, although there were great differences in the initial concentration of TOC in wastewater, the TOC concentration in each bioreactor gradually decreased during the operation of microalgae membrane bioreactor, indicating that glucose in artificial wastewater could be used as carbon and/or energy for microbial mixotrophy growth. For wastewater with an influent TOC concentration of 40 mg/L, 80 mg/L, and 120 mg/L, the effluent TOC concentrations were reduced to 2.01 mg/L, 15.30 mg/L, and 28.30 mg/L, respectively. The TOC removal rate with influent TOC concentrations of 40 mg/L, 80 mg/L, and 120 mg/L could reach 37.99 g/(m^3^·d), 64.70 g/(m^3^·d), and 91.70 g/(m^3^·d), respectively. It could be seen that, although the removal efficiency will be decreased when the TOC concentration is high, the mixotrophy microalgae could also effectively remove most of the organic matter in the wastewater. Therefore, glucose as the carbon source greatly promoted the growth of mixotrophy microalgae.

### 3.3. Effect of Different Influent pH on Microalgae Membrane Bioreactor Performance

#### 3.3.1. *P*. *helgolandica tsingtaoensis* Growth

**Figure 7:**
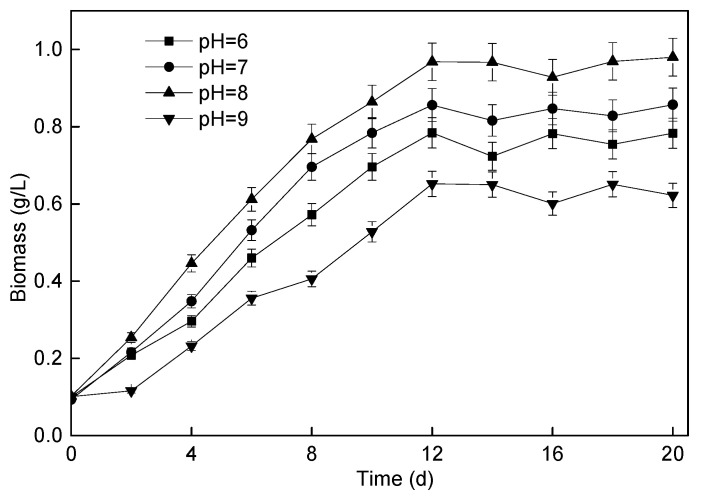
Changes in *P. helgolandica tsingtaoensis* biomass concentration under different influent pH.

In the process of exploring the effect of pH on the operation of microalgae membrane bioreactor, the change in *P. helgolandica tsingtaoensis* biomass was monitored, as shown in Figure 7. It could be seen from the figure that, with the influent pH of 8, the growth of *P. helgolandica tsingtaoensis* biomass was the fastest, and the maximum biomass could reach 0.97 g/L with the *P. helgolandica tsingtaoensis* average growth rate of 70.67 mg/(L·d). For the pH of 6, 7, and 9, the maximum biomasses were 0.78 g/L, 0.86 g/L, and 0.65 g/L, respectively, and the average growth rates of *P. helgolandica tsingtaoensis* were 55.33 mg/(L·d), 61.33 mg/(L·d), and 44.33 mg/(L·d), respectively.

During the operation of the microalgae membrane bioreactor, the pH value was the key and controllable parameter affecting the growth and harvest of microalgae biomass [61]. Studies have shown that the photosynthetic activity of microalgae could significantly increase the pH value [62]. It has been reported that the pH value in the continuous bioreactor was lower than that in the sequencing batch photoreactor, which showed that the continuous culture of microalgae in the reactor was more conducive to the growth of microalgae [63]. However, the optimum pH value of different microalgae was different. For example, when the pH was 6.3–7.5, the yield of *chlorella* in the photobioreactor with dairy wastewater was the highest. Similarly, the yield of *scenedesmus obliquus* was also different at different pH values in the photobioreactor, and the growth rate observed at pH 8 was the best [64].

The experimental results showed that the growth of *P. helgolandica tsingtaoensis* was affected by pH. The influent pH = 8 provided a good growth environment for *Platymonas helgonica tsingtaoensis*, and the growth of *P. helgolandica tsingtaoensis* was inhibited with the influent pH = 9.

#### 3.3.2. Nutrient Removal

**Figure 8:**
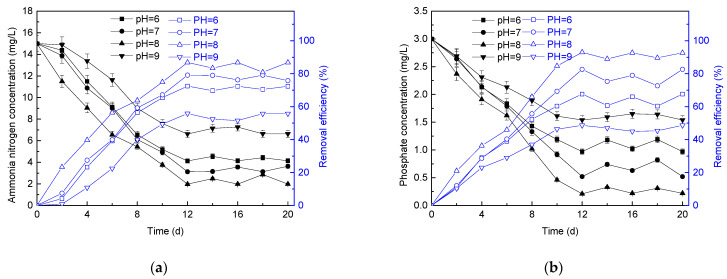
Changes in ammonia nitrogen (**a**) and phosphate (**b**) removal under different pH.

The treatment efficiency of the microalgae membrane bioreactor on ammonia nitrogen is shown in Figure 8a. The experimental group with influent pH of 8 showed a lower effluent ammonia nitrogen concentration compared with those with influent pH of 6, 7, and 9. With the operation of the reactors, the effluent ammonia nitrogen concentration of the reactor with influent pH = 8 could reduce to 1.98 mg/L with the removal efficiency of 87%, and the ammonia nitrogen removal rate could reach 13.03 g/(m^3^·d). With the influent pH of 6, 7, and 9, the effluent ammonia nitrogen concentration could be decreased to 4.13 mg/L, 3.13 mg/L, and 6.63 mg/L, respectively, with the ammonia nitrogen removal efficiency of 72%, 79%, and 56%, respectively, and the ammonia nitrogen removal rate was 10.88 g/(m^3^·d), 11.88 g/(m^3^·d), and 8.38 g/(m^3^·d), respectively.

The removal efficiency of the reactor on total phosphorus is shown in Figure 8b. With the change in pH, the change trend of total phosphorus removal efficiency was similar to that of ammonia nitrogen. It could be seen that, for the influent pH of 6 and 7, the minimum concentration of total phosphorus in the effluent reached 0.97 mg/L and 0.52 mg/L, respectively, with the removal rate of 2.03 g/(m^3^·d) and 2.48 g/(m^3^·d), respectively. For the influent pH of 8, the effluent total phosphorus concentration was as low as 0.11 mg/L, and the removal efficiency reached 93% with the removal rate of 2.79 g/(m^3^·d). With respect to the influent pH of 9, the total phosphorus removal efficiency of the reactor was greatly affected, and the total phosphorus removal rate was only 1.46 g/(m^3^·d).

It had been reported that *P. helgolandica tsingtaoensis* was usually cultured at the suitable pH value of 8.2, and the pH value tended to increase throughout the experiment [21]. Cheng et al. reported that the elevated pH value might result from bicarbonate uptake for photosynthesis [65]. This increase was beneficial for the disinfection of pathogens [21]. Therefore, the pH played an important role in the proliferation of *P. helgolandica tsingtaoensis*, and then had obvious effect on the removal of pollutants by *P. helgolandica tsingtaoensis*.

### 3.4. Effect of Different Influent N/P Ratios on Microalgae Membrane Bioreactor Performance

#### 3.4.1. *P*. *helgolandica tsingtaoensis* Growth

**Figure 9:**
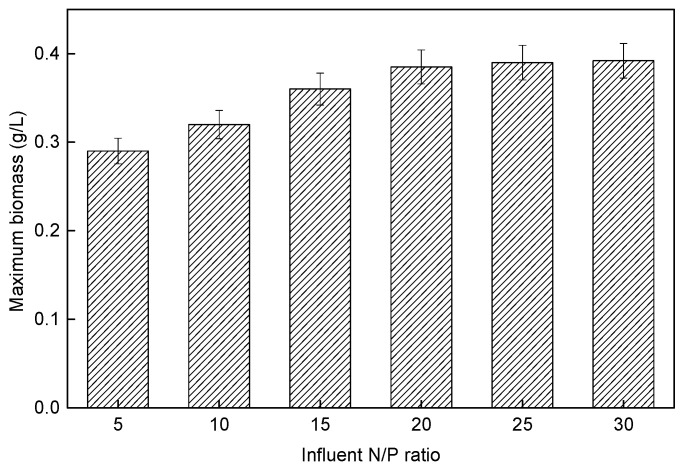
The *P. helgolandica tsingtaoensis* biomass of reactors under different influent N/P ratios at the 20th day.

The biomass of the reactors in each experimental group was measured, as shown in Figure 9. It could be seen that, when the influent N/P ratio increased between 5 and 20, the growth rate of *P. helgolandica tsingtaoensis* increased. When the influent N/P ratio was greater than 20, the growth rate of *P. helgolandica tsingtaoensis* tended to be stable and the maximum biomass was almost the same. For *P. helgolandica tsingtaoensis*, the increase in matrix concentration could promote their absorption of pollutants to a certain extent, in order to promote the growth and reproduction of *P. helgolandica tsingtaoensis*. It was widely accepted that the algal growth rate and field are determined by the abundance of the nutrient [66]. When the influent N/P ratio was low, it might become a limiting factor for the growth of microalgae. Darley (1982) suggested that a higher N/P ratio (e.g., >30) implied a limitation of phosphorus, and lower N/P ratios (e.g., <5) implied a limitation of nitrogen [67]. It was often difficult to quantify nutrient limitation based on a fixed N/P ratio owing to the complexity of phytoplankton community structure and the variation in N/P ratio optima among the different species of micro algae [44]. Therefore, in the actual project, the influent N/P ratio demand of microalgae should be guaranteed to maximize the enrichment of microalgae.

#### 3.4.2. Nutrient Removal

**Figure 10:**
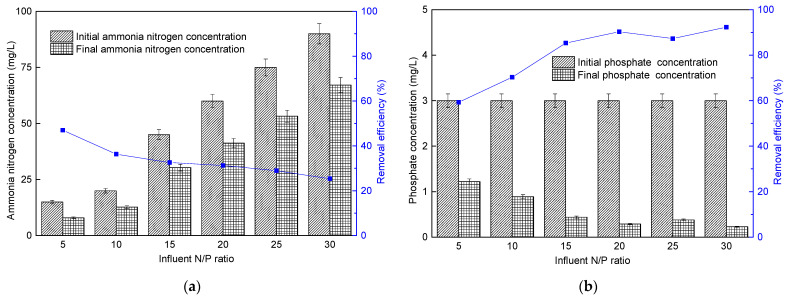
Changes in ammonia nitrogen (**a**) and phosphate (**b**) removal of microalgae membrane bioreactor under different influent N/P ratios at the 20th day.

The ammonia nitrogen removal for the microalgae membrane bioreactor is shown in Figure 10a. When the influent N/P ratio for microalgae membrane bioreactor was 5, 10, 15, 20, 25, and 30, the ammonia nitrogen removal rate was 2.35 g/(m^3^·d), 2.42 g/(m^3^·d), 4.89 g/(m^3^·d), 6.26 g/(m^3^·d), 7.24 g/(m^3^·d), and 7.61 g/(m^3^·d), respectively. Although the influent ammonia nitrogen concentration was inconsistent, it could be seen from the change trend of ammonia nitrogen removal efficiency that the increase in the influent N/P ratio was conducive to the absorption of ammonia nitrogen by microalgae in the reactor. However, when the influent N/P ratio was between 20 and 30, the ammonia nitrogen removal capacity of the reactor tends to be stable, but it would lead to the decline in ammonia nitrogen removal efficiency and affect the effluent quality owing to the increased influent concentration.

The total phosphorus concentration and removal efficiency of microalgae membrane bioreactor are shown in Figure 10b. When the influent N/P ratio increased from 5 to 20, the total phosphorus removal rates increased from 0.59 g/(m^3^·d) to 0.90 g/(m^3^·d), and the removal efficiency increased by 52%. When the influent N/P ratio was greater than 20, the total phosphorus removal rate tended to be stable, which was roughly the same as the change trend of ammonia nitrogen and biomass.

The ratio of nitrogen/phosphorus (N/P) was known to affect the cell proliferation of *P. helgolandica tsingtaoensis*. Many previous studies have indicated that microalgae assimilation was the primary manner of nutrient removal [8]. The ability to transform and utilize nitrogen varies among micro algae, resulting in differences in algal demands for N/P ratio in coastal waters [44]. When N/P increased from 5 to 20, the growth rate and pollutant removal efficiency of *P. helgolandica tsingtaoensis* increased gradually; when N/P is higher than 20, the pollutant removal efficiency of the reactor tends to be stable and does not change with the increase in N/P.

## 4. Conclusions

In this study, *Platymonas helgonica tsingtaoensis* was used as an algae species to treat simulated mariculture wastewater by internal circulating fluidized bed microalgae membrane bioreactor. The nutrient removal efficiency and biomass production of microalgae membrane bioreactor under different influent conditions were discussed. The results showed that, compared with the autotrophic and heterotrophic modes, the highest *P. helgonica tsingtaoensis* growth rate and nutrient removal efficiencies were observed in the mixotrophy culture mode. The *P. helgonica tsingtaoensis* growth and nutrient removal efficiencies could be significantly promoted with the increase in influent organic matter concentration. Among the different influent pH values, the *P. helgonica tsingtaoensis* biomass at the influent pH of 8 increased fastest, and the nutrient removal efficiencies were the highest. With the influent N/P ratio increasing from 5 to 20, the *P. helgonica tsingtaoensis* growth rate and nutrient removal rate increased gradually. When the influent N/P ratio was higher than 20, the nutrient removal efficiency of the reactor tended to be stable and did not change with the increase in the influent N/P ratio. At the proper influent conditions, high biomass and nutrient removal efficiency could be obtained in the microalgae membrane bioreactor, which was a suitable object for further practical application for wastewater treatment.

## Figures and Tables

**Table 1:** Experimental arrangement for studying the treatment efficiency of and biomass production of the microalgae membrane bioreactor under different operating conditions.

System Parameters	Culture Modes	Influent TOC (mg/L)	pH	Influent Ammonia (mg/L)	Influent Phosphate (mg/L)	Operation Time (days)	HRT	Flux (L/m^2^·h)	Temperature (°C)
Run 1	Photoautotrophic	60	7.5	15	3	20	1	3.9	25
	Heterotrophic								
	Mixotrophy								
Run 2	Mixotrophy	40	7.5	15	3	20	1	3.9	25
		80							
		120							
Run 3	Mixotrophy	120	6.0	15	3	20	1	3.9	25
			7.0						
			8.0						
			9.0						
Run 4	Mixotrophy	120	8.0	15	3	20	3	1.3	25
				30					
				45					
				60					
				75					
				90					
